# Adaptation to fluctuating environments in a selection experiment with *Drosophila melanogaster*

**DOI:** 10.1002/ece3.2965

**Published:** 2017-04-18

**Authors:** Olga I. Kubrak, Sören Nylin, Thomas Flatt, Dick R. Nässel, Olof Leimar

**Affiliations:** ^1^ Department of ZoologyStockholm UniversityStockholmSweden; ^2^ Department of Ecology and EvolutionUniversity of LausanneLausanneSwitzerland

**Keywords:** experimental evolution, food intake, generalist phenotype, reaction norm, resource storage, starvation resistance

## Abstract

A fundamental question in life‐history evolution is how organisms cope with fluctuating environments, including variation between stressful and benign conditions. For short‐lived organisms, environments commonly vary between generations. Using a novel experimental design, we exposed wild‐derived *Drosophila melanogaster* to three different selection regimes: one where generations alternated between starvation and benign conditions, and starvation was always preceded by early exposure to cold; another where starvation and benign conditions alternated in the same way, but cold shock sometimes preceded starvation and sometimes benign conditions; and a third where conditions were always benign. Using six replicate populations per selection regime, we found that selected flies increased their starvation resistance, most strongly for the regime where cold and starvation were reliably combined, and this occurred without decreased fecundity or extended developmental time. The selected flies became stress resistant, displayed a pronounced increase in early life food intake and resource storage. In contrast to previous experiments selecting for increased starvation resistance in *D. melanogaster*, we did not find increased storage of lipids as the main response, but instead that, in particular for females, storage of carbohydrates was more pronounced. We argue that faster mobilization of carbohydrates is advantageous in fluctuating environments and conclude that the phenotype that evolved in our experiment corresponds to a compromise between the requirements of stressful and benign environments.

## 1 Introduction

How organisms cope with fluctuating environments is one of the main questions in life‐history evolution (Houston & McNamara, 1999; Levins, 1968; Roff, 1992; Saether & Engen, 2015; Stearns, 1976; Tuljapurkar, Gaillard, & Coulson, 2009). An important category of environmental fluctuation is when different generations of a short‐lived organism encounter substantially different conditions. In cases where environmental cues are available and accurately predict near‐future environments, phenotypic plasticity and transgenerational effects are possible evolutionary outcomes (Dey, Proulx, & Teotónio, 2016; Flatt, Amdam, Kirkwood, & Omholt, 2013; Moran, 1992; Rueffler, Van Dooren, Leimar, & Abrams, 2006). For unpredictable fluctuations, where reliable cues are unavailable, two main types of evolutionary responses have been proposed: a compromise, generalist phenotype that performs reasonably well in all environments, or a spectrum of phenotypes, referred to as diversified bet‐hedging (Donaldson‐Matasci, Lachmann, & Bergstrom, 2008; Hopper, 1999; Leimar, 2005; Saether & Engen, 2015; Seger & Brockmann, 1987; Simons, 2011).

Here, we have performed a selection experiment where we exposed a wild‐derived population of *Drosophila melanogaster* to experimental evolution in the laboratory (Kawecki et al., 2012), to examine the evolutionary responses to fluctuation between stressful and benign conditions. The stressor we imposed consisted of 3–4 days of starvation, which was calibrated to cause a mortality of about 50% at the onset of the experiment. In addition, we imposed a (reliable or unreliable) cue of upcoming starvation, in the form of a 2‐hr cold shock at 0°C early in adult life. This cue did not cause direct mortality but nevertheless might act at a stressor by reducing survival or reproduction over the life cycle.

Responses to selection for starvation resistance in *D. melanogaster* have been studied previously (Hoffmann & Harshman, 1999; Rion & Kawecki, 2007; Schwasinger‐Schmidt, Kachman, & Harshman, 2012), and a main conclusion from this work is that starvation resistance is robustly associated with a “survival mode” phenotype, entailing longer larval developmental time, larger adult body mass and energy reserves, lower fecundity, and longer lifespan, possibly mediated by insulin signaling (Hansen, Flatt, & Aguilaniu, 2013; Rion & Kawecki, 2007). With respect to energy reserves, the most consistent finding is a positive correlation between starvation resistance and increased lipid reserves in adult flies (Chippindale, Chu, & Rose, 1996; Djawdan, Chippindale, Rose, & Bradley, 1998; Harshman, Hoffmann, & Clark, 1999; Hoffmann & Harshman, 1999; Masek et al., 2014), but higher carbohydrate reserves have also been found (Djawdan et al., 1998). One suggestion is that altered lipid metabolism is most important during starvation, whereas carbohydrate metabolism dominates during exposure to desiccation (Marron, Markow, Kain, & Gibbs, 2003). The rate at which resources can be mobilized is another potentially important factor, in particular in unpredictably fluctuating environments. In general, carbohydrate reserves have a more rapid turnover than lipids (Lee & Jang, 2014; Wigglesworth, 1949).

Although experiments with alternating, or otherwise temporally varying, selection have previously been performed with *D. melanogaster* (e.g., Kellermann, Hoffmann, Kristensen, Moghadam, & Loeschcke, 2015; Manenti, Loeschcke, Moghadam, & Sorensen, 2015), especially in connection with the study of phenotypic plasticity (Scheiner, 2002), previous selection experiments on starvation resistance in *D. melanogaster* have used a design that corresponds to a predictable environment. This might have important consequences for the responses seen in these experiments. For instance, it has been suggested that the association between lipid reserves and starvation resistance seen in these studies is a result of the imposed laboratory conditions and is not detected when examining variation among wild‐derived inbred lines (Jumbo‐Lucioni et al., 2010).

**Figure 1:**
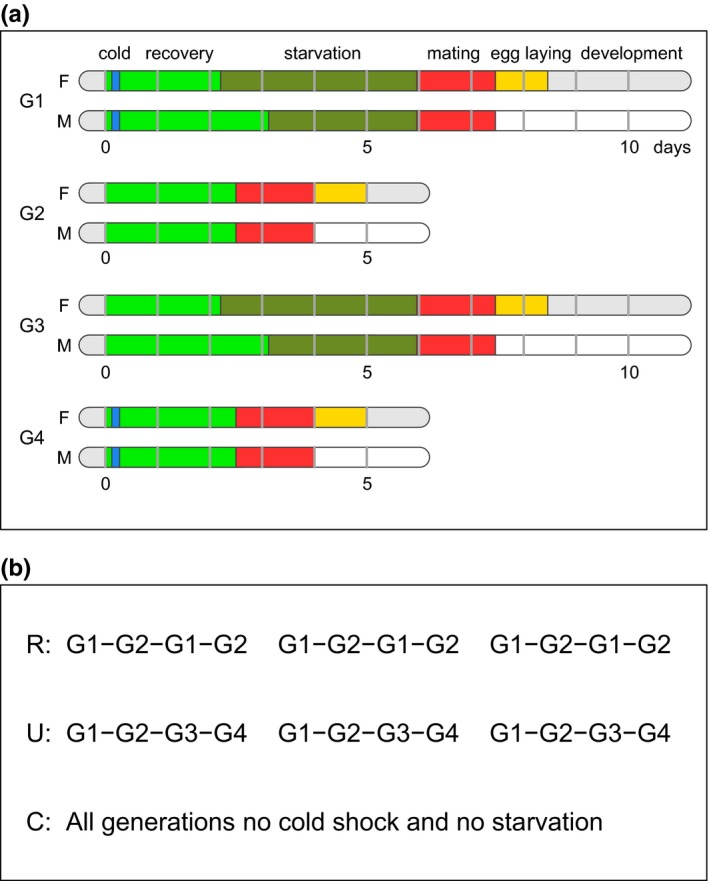
Illustration of the experimental design. (a) Four kinds of generations, labeled G1, G2, G3, G4, for females, F, and males, M, were used to construct the selection regimes. The horizontal bars show the progress of time, with the zero point corresponding to collection of newly hatched adults. The color coding indicates the conditions imposed: Blue is cold shock, green is food ad lib (recovery from cold shock), dark green is starvation, red is mating, yellow is egg laying (both with food ad lib), and light gray is development from egg toward adult hatching. (b) The selection regimes are defined by different sequences of generations from the ones shown in (a), arranged in three blocks of four generations each, making a total of 12 generations. Long generations with starvation are alternated with short generations without starvation. In the R regime, cold shock and starvation are reliably linked, whereas in the U regime, they are uncorrelated. The C regime is a control without any cold shock or starvation. There were six replicate selection lines for each selection regime with at least 500 flies in every replicate, 250 females and 250 males

In our experiment, we alternated between starvation and benign conditions over generations. In one selection regime (R), generations with starvation were always initiated with a cold shock; thus, in principle acting as a reliable cue of the ensuing starvation, and in another regime (U), flies were exposed to starvation in the same way but cold shock and starvation were uncorrelated (Figure 1). Under these conditions, there should be an advantage in surviving and maintaining reproductive capacity during a several‐day long period of starvation if starvation occurs. However, it is also advantageous to be able to mobilize resources for early reproduction if there is no starvation. Among the possible evolutionary outcomes is a norm of reaction encompassing cold shock‐induced starvation resistance in the R regime and adaptation to unpredictably occurring cold shock and starvation stressors in the U regime. In the event that there is little standing genetic variation for a cold shock—starvation resistance reaction norm in the base population, regime R might also result in adaptation to unpredictably occurring cold shock and starvation stress.

Based on previous work on selection for starvation resistance in *D. melanogaster*, the predicted outcome for selection regime R, where cold shock reliably indicates upcoming starvation, would be a reaction norm where cold shock induces a “survival mode” phenotype, with larger adult body mass and energy reserves, in particular greater lipid storage, and with lower fecundity. The absence of cold shock, on the other hand, would be predicted to induce a phenotype suited to benign conditions. In our experiment, there was a third selection regime where conditions were benign in each generation (Figure 1), which we refer to as control (C). Finally, for selection regime U, the predicted outcome of selection would either be a generalist phenotype, intermediate between a “survival mode” phenotype and one suited to benign condition, or a risk‐spreading spectrum of phenotypes.

After applying the selection regimes, we scored the phenotypes of the flies by measuring a large number of fitness‐relevant traits, including starvation resistance, female fecundity, egg‐to‐adult developmental time and survival, chill‐coma recovery, oxidative and desiccation stress resistance, feeding behavior, body size, and energy reserves. We interpret our results in light of our predictions and compare them with previous selection experiments on starvation resistance with constant environments.

## 2 Materials and Methods

### 2.1 Base population and maintenance

The flies used in the experiment were derived from several hundred female *D. melanogaster* that were wild caught in 2007 in a vineyard near Sierre in the Canton Valais, Switzerland (kindly donated by Tadeusz Kawecki, Department of Ecology and Evolution, University of Lausanne). They were maintained at a large population size and were used in the study by Nepoux, Haag, and Kawecki (2010), where more details can be found. The base population for our experiment (1250 flies) was propagated and kept on an enriched medium containing 100 g/L sucrose, 50 g/L yeast, 12 g/L agar, 3 ml/L propionic acid, and 3 g/L nipagin. Experimental flies were grown under uncrowded conditions at 25°C and a 12L:12D photoperiod.

### 2.2 Design of selection experiment

The selection experiment was performed from October 2014 to June 2015, with a total of 18 replicate populations (lines), split into three selection regimes (R, U, C; see Figure 1) with six replicate lines each and at least 500 flies in each replicate (250 females and 250 males), making up a total of more than 9000 flies maintained during the experiment. The replicate lines were run in parallel, with alternating long and short generations, where long generations included a period of starvation for selection regimes R and U (Figure 1a). Starvation was longer for females than males in order to achieve similar starvation mortality for the sexes (females of *D. melanogaster* have on average higher starvation resistance than males), with the duration calibrated to achieve around 50% mortality at the start of the experiment. Females and males were separated when they were collected as newly eclosed (3–6 hr old) unmated adults and were kept separated until the time of mating (Figure 1a). The regimes R and U consisted of 3 blocks of 4 generations (Figure 1b), giving a total of 12 generations of selection. Each block implemented either reliable coupling between cold shock and starvation (regime R), in which case cold shock acted as a potential cue of the coming starvation, or no correlation between them (regime U; Figure 1b). We chose cold shock as a stressor that might act as a cue because previous studies have found a relation between cold and starvation resistance (Bubliy & Loeschcke, 2005; Hoffmann, Hallas, Anderson, & Telonis‐Scott, 2005). Both before and after the selection experiment, a number of traits relating to life history and physiology were measured and analyzed using mixed‐model statistical approaches, including Bayesian MCMC inference for censored survival data.

### 2.3 Trait measurements

The measurements were performed at 25°C and 12L:12D, generating data for each of the 18 replicate lines. See below for details on sample sizes. Starvation survival (no food, but with water supplied as 0.5% agarose), and fecundity of 4, 7, and 14 days, old females were measured in generation 14. Egg‐to‐adult developmental time and survival were measured in generation 19. A number of measures of stress resistance and physiology were recorded for 4‐ to 5‐day‐old unmated flies of both sexes, after 12 generations of selection for flies from regimes R and U, and before the start of the selection experiment for control flies (C). These included chill coma recovery after 2 h at 0°C, desiccation survival (without access to water or food), and oxidative stress survival (on the enriched food medium, supplemented with 20 mM paraquat[methyl viologen], 856177: Sigma‐Aldrich). A capillary feeding (CAFE) assay was performed according to Ja et al. (2007) with 5 μl capillaries filled with food composed of 50 g/L sucrose, 50 g/L yeast, and 3 ml /L propionic acid. The food consumption was recorded every 24 h with refilling of capillaries. We also measured body weight, water content, concentrations of circulating (hemolymph) glucose, together with stored (whole body) glucose, trehalose, and glycogen as well as stored lipids (triacylglycerides, TAG). All assays were performed according to (Tennessen, Barry, Cox, & Thummel, 2014) as described in detail in (Kubrak, Kučerová, Theopold, & Nässel, 2014).

**Figure 2:**
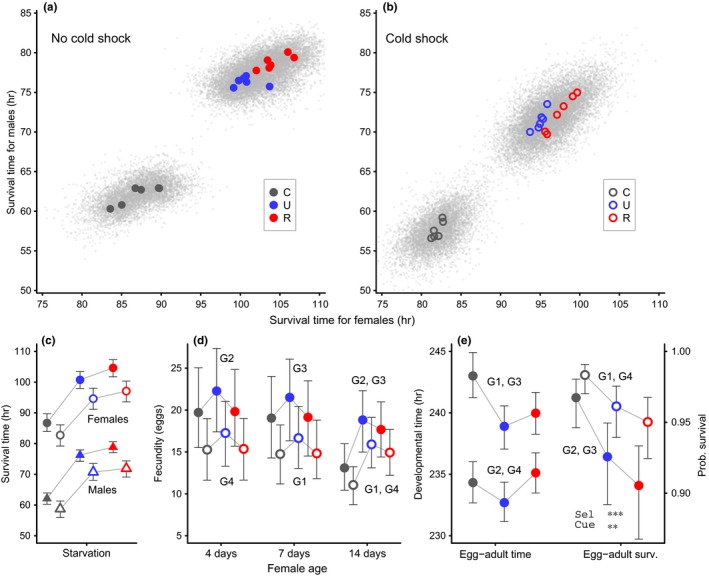
Life‐history characteristics of the selection regimes after the end of selection (generation 14). The top panels show Bayesian MCMC estimated duration of survival under starvation for females and males of the six replicates for each of the selection regimes, C, U, and R. The color‐coded points show the estimated replicate male versus female mean survival without cold shock (a, filled symbols) and with cold shock (b, open symbols). The clouds of fine gray points show the overall distribution of posterior survival times from the model output. The top and bottom panels use the same coding. (c) Bayesian means and 95% confidence intervals for the posterior distributions shown in the top panels; see also Table 1. (d) Bayesian means and 95% confidence intervals for the number of eggs laid per day for the selection regimes. Left and middle show egg laying during one day of the mating period in generations G1 to G4 from Figure 1. Right shows fecundity at 14 days of age for these generations; see also Table 2. (e) Characteristics of egg‐to‐adult development for the selection regimes, for eggs laid in generations G1 to G4 from Figure 1. Left shows Bayesian means and 95% confidence intervals for egg‐to‐adult developmental time (no statistically significant effect of cold shock, see also Table 3) and right shows mean and 95% confidence intervals of egg‐to‐adult survival (no statistically significant effect of starvation). A simplified ANOVA table giving statistical significances is shown at bottom right in the plot (Sel is selection regime, Cue is cold shock)

**Figure 3:**
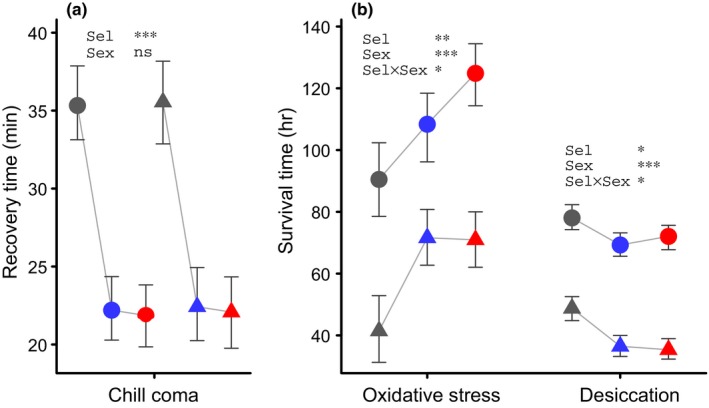
Reaction to stressors for 4‐ to 5‐day‐old flies from the selection regimes, before the start of the selection experiment for regime C, and after 12 generations of selection for regimes U and R. (a) Chill coma recovery after a cold shock of 2 h at 0°C. (b) Survival under oxidative stress (left) and desiccation stress (right). Color coding indicates selection regimes C, U, and R and the shape of symbols (round, F, and triangle, M) the two sexes. Data are given as Bayesian means and 95% confidence intervals. Simplified ANOVA tables giving statistical significances are shown in the plot (Sel is selection regime)

**Figure 4:**
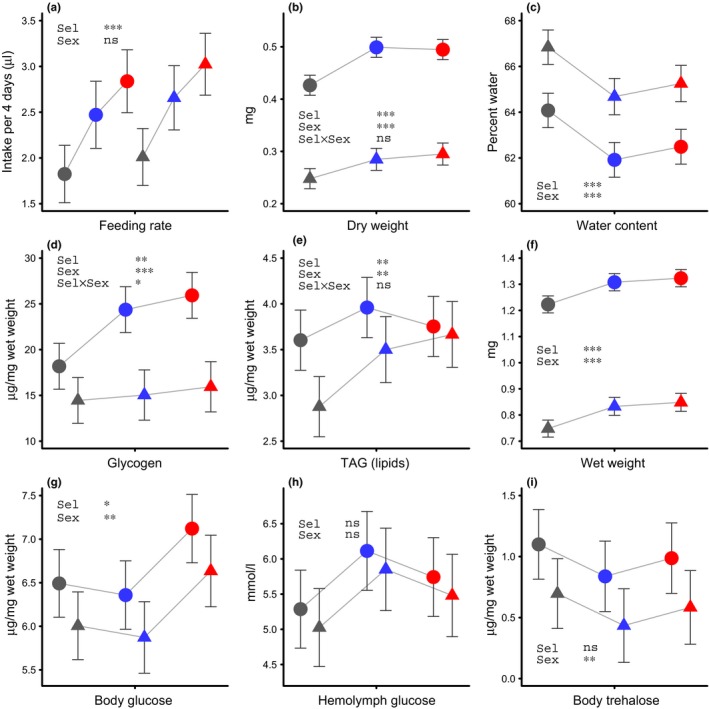
Feeding, body weight, and resource storage for 4‐ to 5‐day‐old flies from the selection regimes, before the start of the selection experiment for regime C, and after 12 generations of selection for regimes U and R. Plotting symbols are as in Figure 3. (a) Accumulated feeding over 4 days of life. (b–i) show dry weight, percentage water of the wet weight, wet weight, and concentrations of glycogen, triacylglycerol (TAG), body glucose, hemolymph glucose, and body trehalose. Data are shown as means and 95% confidence intervals obtained from the statistical model fitting. Simplified ANOVA tables giving statistical significances are shown in the plot (Sel is selection regime)

The sample sizes for the different measurements, presented in the order they appear in the results section, were as follows. For starvation survival (Figure 2a–c; Table 1), females and males were placed in separate vials, with 220 vials with an average of 19.8 females per vial and 215 vials with an average of 18.2 males per vial, making up a total of 8248 flies for this measurement. For female fecundity (Figure 2d; Table 2), 339 females were used for the number of eggs laid at ages 7 and 4 days (159 + 180), and 312 females were used for the number of eggs laid at age 14 days. This means that somewhat less than five females were used per combination of replicate line, cold shock cue, and starvation treatment. For egg‐to‐adult developmental time and survival (Figure 2e; Table 3), newly laid eggs, for which the parents had experienced the requisite cold shock and starvation treatments, were placed in vials and, as they developed, it was recorded in which time interval they eclosed from the pupa, or if they failed to eclose. There were 295 vials with an average of 7.6 individuals per vial, giving a total of 2228 individuals. For chill coma recovery (Figure 3a), females and males were placed in separate vials, with 33 vials with an average of 15.3 females per vial and 33 vials with an average of 14.5 males per vials, making up a total of 983 flies for this measurement. For oxidative stress survival (Figure 3b), females and males were placed in separate vials, with 41 vials with an average of 21.5 females per vial and 50 vials with an average of 21.0 males per vials, making up a total of 1931 flies for this measurement. For desiccation survival (Figure 3b), females and males were placed in separate vials, with 57 vials with an average of 16.9 females per vial and 52 vials with an average of 15.0 males per vials, making up a total of 1743 flies for this measurement. For feeding rate (Figure 4a), the intake over 4 days was measured for 84 females and 92 males, which corresponds to 4.7 females and 5.1 males per replicate line. For each of dry weight, wet weight, water content, and other physiological variables (Figure 4b–i), there was one measurement for females and males of each replicate line. Each such measurement is an average based on 15–20 flies measured as a group and dividing with the number of flies in the group or the wet weight of the group, so for each variable 500–600 flies were used.

**Table 1:** Bayesian MCMC model output for the number of hours of starvation survival, estimated in generation 14

Effect	Estimate	95% C.I.
Intercept	104.64	(101.79, 107.44)
Sex M	**−**25.80	(**−**28.78, **−**22.92)
Sel U	**−**3.89	(**−**7.81, **−**0.23)
Sel C	**−**17.92	(**−**21.66, **−**14.23)
Cue Cs	**−**7.56	(**−**10.26, **−**4.88)
Sel C × Cue Cs	3.58	(0.18, 6.85)

The intercept represents females (F) from selection regime R without cold shock. The table gives the statistically significant effects of the factors sex (Sex M), selection regime (Sel U, Sel C), cold shock cue (Cue Cs), and their interaction. See Appendix 1 for more details.

**Table 2:** Output from Bayesian MCMC negative binomial regression for the number of eggs laid by females during one day (18 h) in generation 14, as illustrated in Figure 2d

Variable	Effect	Estimate	95% C.I.
Early fecundity (4 or 7 days)	Intercept	2.97	(2.72, 3.21)
	Sel U	0.12	(**−**0.16, 0.39)
	Sel R	**−**0.01	(**−**0.26, 0.28)
	Cue Cs	**−**0.26	**(−0.45, −0.06)**
	Starv S	**−**0.04	(**−**0.26, 0.18)
Mid‐life fecundity (14 days)	Intercept	2.56	(2.35, 2.78)
	Sel U	0.36	**(0.12, 0.60)**
	Sel R	0.30	**(0.04, 0.53)**
	Cue Cs	**−**0.17	(**−**0.33, 0.01)
	Starv S	0.01	(**−**0.18, 0.20)

The intercept corresponds to selection regime C, without cold shock, and without starvation (early fecundity at 4 days old, mid‐life fecundity at 14 days old), and represent log expected number of eggs. The table gives effects of selection regime (Sel U, Sel R), cold shock (Cue Cs), and starvation (Starv S, early fecundity at 4 or 7 days old, mid‐life fecundity at 14 days old). Confidence intervals entailing statistically significant effects are shown in bold.

**Table 3:** Bayesian MCMC model output for the number of hours of egg‐to‐adult developmental time, estimated in generation 19

Effect	Estimate	95% C.I.
Intercept	235.12	(233.52, 236.80)
Sel U	**−**2.43	**(−4.56, −0.15)**
Sel C	**−**0.80	(**−**3.13, 1.48)
Starv S	4.86	**(2.77, 6.73)**
Sel U × Starv S	1.35	(**−**1.44, 4.40)
Sel C × Starv S	3.83	**(0.78, 6.80)**

The intercept represents individuals (both sexes together) from selection regime R without cold shock. The table gives effects of selection regime (Sel U, Sel R), starvation (Starv S), and interactions of selection regimes U and C with starvation. Confidence intervals entailing statistically significant effects are shown in bold.

### 2.4 Statistical methods

We used the R statistical software (R Core Team 2016). In the mixed‐model analyses, selection regime (Sel) and, where relevant, sex (Sex) were always included as fixed effects, and population replicate line (Repl) as a random effect. Other fixed‐effect factors, depending on the trait analyzed, were cold shock (Cue) and starvation (Starv), which were implemented as in the selection experiment (Figure 1a). For starvation survival, we also controlled for the precise age of adults at the time starvation started, as it has previously been found that starvation resistance can change with time after adult emergence in *D. melanogaster* (Chippindale et al., 1996). For “time‐to‐event” traits (i.e., survival traits, developmental time, and chill coma recovery), measurements were taken on flies grouped together in vials, and for these “vial” was included as a random effect.

For survival traits, including starvation survival, the raw measurements consisted of counts of surviving and dead flies in each vial at a specified number of time points, which means that observations of survival time, viewed as a response variable, are interval censored, that is, appear as counts of the flies in a vial that died in a particular time interval. Furthermore, for practical reasons, there was variation in the length of the intervals (e.g., longer time interval overnight). To perform a proper mixed‐model analysis for this situation, we used the Bayesian MCMC Stan framework (Stan Development Team 2015) to analyze statistical models of survival time. In these models, the random effects were assumed to be normally distributed. Our statistical analysis is novel and overcomes some of the restrictions on censoring and mixed effects in common approaches to survival analysis. Chill‐coma recovery and egg‐to‐adult developmental time were analyzed in the same way. More details on our approach appear in Appendix 1, and the data for the analysis and the R and Stan scripts are deposited at the Dryad data repository (doi:10.5061/dryad.4790c).

For other response variables, we used the lmer function in the R package lme4 (Bates, Maechler, Bolker, & Walker, 2015) to fit mixed‐effect models, except for fecundity where we used the stan_glmer function in the rstanarm package (Gabry & Goodrich, 2016), with a negative binomial family in order to allow for overdispersion in the number of eggs laid by a female, and for egg‐to‐adult survival where we used the glmer function in the lme4 package with a binomial family.

## 3 Results

Over the course of the experiment, the flies from the uncorrelated (U) and reliable (R) selection regimes increased their survival over the set starvation period, starting out at around 50% survival and reaching around 90%. The statistical analysis of starvation survival after the experiment showed that flies from regime R survived about 4 hr longer than flies from regime U and about 18 h longer than those from regime C (Table 1; Figure 2a–c). Cold shock given before starvation reduced starvation survival by about 8 hr for regime R and U, and by about 4 h for the control (C) regime (Table 1). We did not find that flies from regime R, as compared to regime U, responded to cold shock by increased starvation resistance; if anything, the R–U survival difference was smaller with cold shock than without (Figure 2a, b). Thus, the R flies did not evolve the predicted norm of reaction. The analysis of female fecundity after the experiment showed that cold shock reduced fecundity; however, selected females (R and U) did not have lower fecundity than control females (C) at the age they laid eggs in the selection experiment (Figure 2d; Table 2). For 14‐day‐old females, which is around the age where *D. melanogaster* females typically have a high rate of egg laying, the fecundity was higher for selected females (R and U) than controls (C) (Figure 2d; Table 2), in disagreement with the existence of a starvation resistance—fecundity trade‐off. The analysis of egg‐to‐adult developmental time also failed to reveal a trade‐off with starvation resistance, as selected flies displayed similar or slightly shorter developmental time (Figure 2e; Table 3). For egg‐to‐adult survival, on the other hand, selected flies displayed lower survival than controls (Figure 2e), consistent with a trade‐off.

In addition to increased starvation resistance, selected flies also improved their resistance to other stressors by gaining more rapid recovery from chill coma (Figure 3a) and extended survival under oxidative stress induced by feeding paraquat (Figure 3b). Survival during desiccation (dry starvation), however, was decreased in selected flies as compared to controls (Figure 3b). These effects might be a consequence of modifications of behavior and physiology of the selected flies. In R flies in particular, but also in U flies, food intake early in life was considerably higher than in C flies (Figure 4a), and at 4‐5 days of age they exhibited higher dry (Figure 4b) and wet weight (Figure 4f), but a lower water content than control flies (Figure 4c). Next we monitored circulating and stored carbohydrates and lipids (triacylglycerides, TAG) in the experimental flies. Selected females stored more glycogen than controls (Figure 4d), but there was no statistically significant difference for males. Selected males had higher levels of TAG (Figure 4e), but there was no statistically significant increase for females. There was a less pronounced higher concentration of glucose in R regime flies (Figure 4g).

## 4 Discussion

Our selection experiment with wild‐derived *D. melanogaster* generated a strong evolutionary response in terms of increased starvation resistance (Figure 2a–c) over 12 generations, together with increased adult body mass and resource storage (Figure 4), indicating substantial amounts of initial standing genetic variation for these traits. Our study is the first to attempt to select for a norm of reaction of increased starvation resistance in response to a reliable environmental cue (cold shock) in fluctuating environments but—contrary to our prediction—no such norm of reaction evolved (Figure 2a, b). Instead, the combination of cold shock and starvation appeared simply to act as a stronger stressor, with a stronger response in the R than the U selection regime (Figure 2a, b; Table 1). Thus, it seems that the cold shock, which did not cause direct mortality, had the effect of lowering starvation resistance. The R flies always experienced cold shock prior to starvation, so on average, they were exposed to more severe starvation stress than the U flies.

Our finding of increased starvation resistance, but no adaptive plasticity in response to the cold shock cue, might be expected if there was initially a large amount of standing genetic variation for starvation resistance, but less, or no, standing genetic variation around a presumed cold shock—starvation resistance reaction norm. In the latter case, it remains possible that perhaps more generations would be required to select for such a reaction norm (see also Chevin & Lande, 2015).

Our finding of a substantially greater starvation resistance in selected flies is in good agreement with previous work on selection for starvation resistance in *D. melanogaster* (see Rion & Kawecki, 2007; and references therein). Previous studies have selected for starvation resistance in every generation, but we alternated stressful and benign conditions between generations (Figure 1), and this lead to a different evolutionary outcome. For example, we did not find correlated responses to selection for starvation resistance in terms of reduced fecundity or extended egg‐to‐adult developmental time, but rather tendencies in the opposite direction (Figure 2d, e). Another difference from previous work, for instance, by Schwasinger‐Schmidt et al. (2012), is that increases in resource storage in our study were not dominated by lipids (TAG). For males, we found increased lipids in selected flies (Figure 4e), but for females, we instead found increased glycogen stores (Figure 4d). Thus, the phenotype that evolved in our selected flies was not simply an intermediate between that of unselected flies and the phenotype that was previously found in experiments with starvation imposed in every generation, which means that our results deviated from the prediction for the U selection regime.

Harshman and Schmid (1998) put forward the idea that selection for starvation resistance should result in increased storage of different resources in proportion to how much each resource was consumed during starvation. This was tested by Schwasinger‐Schmidt et al. (2012), but that study did not fully support the idea. They found that both TAG and glycogen were consumed during starvation, but only TAG increased as a response to selection for starvation resistance. A possible explanation might be differences in energy efficiency among stored compounds (measured as energy per weight of storage), but also in how rapidly they can be mobilized. In a classic study on resource utilization in *D. melanogaster*, Wigglesworth (1949) noted that lipids and glycogen were consumed during starvation in proportion to their availability, but that only glycogen was used during flight, and he attributed this to the comparatively slow rate of lipid metabolism. On the other hand, TAG is more efficient than glycogen in terms of energy density (Djawdan, Sugiyama, Schlaeger, Bradley, & Rose, 1996; Lee & Jang, 2014), which could explain why lipid storage is beneficial in terms of starvation resistance, while the faster mobilization of glycogen storage makes it more suitable for demanding activities like short term flight, and presumably also for mating and egg laying. We therefore suggest that the differences between our results and previous ones with respect to lipid storage are a consequence of the design of the stress exposure protocol. In our design, there is variation in whether a given generation faces starvation, with an ensuing need for stored resources, or early reproduction, in which case, there may be a need for rapid resource mobilization, in particular for females.

A number of studies have compared starvation resistance and resource storage between *Drosophila* populations along a latitudinal cline or from different geographic regions, with mixed results. A relationship between starvation resistance and lipid content was discovered in some cases (Goenaga, Fanara, & Hasson, 2013) but not all (Hoffmann, Hallas, Sinclair, & Mitrovski, 2001). In other cases, starvation resistance was found to be associated with glycogen content (Jumbo‐Lucioni et al., 2010), or with both lipid and glycogen content (Aggarwal, 2014). The reason for these contrasting findings remains unclear, but the various differences in the efficiency of storage compounds mentioned above might be important.

The strongly increased rate of early life food intake we observed in the selected flies in our experiment (Figure 4a) is likely to have caused increased body mass and resource storage. Higher rates of feeding could potentially impose a mortality cost in the wild, due to increased exposure to predation and parasitism, and thus generate a trade‐off with starvation resistance in the wild, but not under laboratory conditions. From what is known about the regulation of feeding in *D. melanogaster* adults (Albin et al., 2015; Itskov et al., 2014; Pool & Scott, 2014), it is not clear what induced the changes in feeding behavior in our selected flies. The reduction in water content we observed in selected flies (Figure 4c) could be a response to selection for increased resource storage, by making the increase in dry weight (Figure 4b) somewhat greater in comparison with the increase in wet weight (Figure 4f). Reduced water content might explain the reduced desiccation resistance in selected flies (Figure 3b), and this could increase mortality in the wild (Gibbs, Chippindale, & Rose, 1997), generating a trade‐off with starvation resistance.

It is a common observation that organisms evolve along an axis of life‐history variation, ranging from a “reproductive mode” (increased reproduction or growth, at the expense of somatic maintenance and survival) at one end to a “survival mode’ (increased somatic maintenance, stress resistance and survival, at the expense of reproduction or growth) at the other end (e.g., Flatt et al., 2013; Rion & Kawecki, 2007; Stearns, 1992). The resulting evolutionary trade‐off between the reproductive and survival modes could be due to tight negative correlations among life history and physiological traits, perhaps as a result of antagonistic pleiotropy. On the other hand, there is growing evidence that this trade‐off is not absolute and can be “de‐coupled” (Flatt, 2011), which suggests that under some circumstance, the traits that contribute to either the reproductive or the survival mode can vary and evolve independently (e.g., Ayroles et al., 2009). Our results lend some support to this latter alternative, in that our selected flies evolved a combination of “survival mode” traits with “reproductive mode” traits.

We conclude that the outcome of our selection experiment, imposing fluctuating selective environments, is a compromise, generalist phenotype, performing reasonably well in all environments encountered. This phenotype is, however, not simply an intermediate between the phenotypes that would evolve in constant benign and constant stressful environments. A generalist must be intermediate if adaptation would occur in a single trait, but if several traits contribute to adaptation, a generalist might instead display different trait combinations than specialists. The phenotype that evolved in the selected flies in our experiment seems to consist of changes in several traits, including increased early adult feeding with a sequestering of resources suitable both for starvation resistance and early reproduction. This phenotype might entail compromises, for instance, between efficiency in terms of energy concentration and speed of resource mobilization. Such trait combinations might be a common way of achieving a generalist strategy in nature. A selection experiment involving fluctuating environments, as in our work here, is one way of establishing instances of such generalist phenotypes.

## Conflict of Interest

None declared.

## Data Archiving

Data and R and Stan files used for the statistical analyses are deposited in the Dryad Repository (doi:10.5061/dryad.4790c).

## Authors’ Contributions

All authors took part in the design of the study and interpreted the results, OK performed the selection experiment and trait measurements, OL performed the statistical analyses and wrote the manuscript, with modifications provided by the other authors.

## Figures and Tables

**Figure A1:**
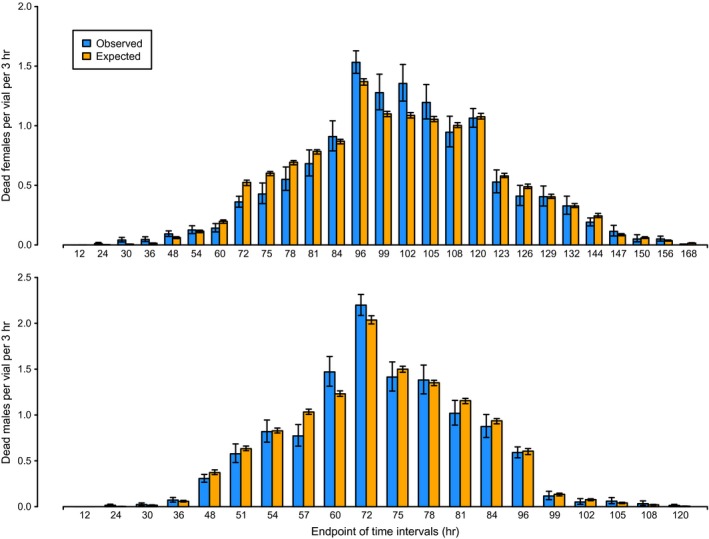
Posterior predictive check for the starvation survival analysis. The panels show the observed and model predicted number of females (top) and males (bottom) per 3 hr that died in each time interval. The first interval starts at 0 hr and ends at 12 hr, the second starts at 12 hr and ends at 24 hr, etc. The values are averages over all vials for the respective sex. The error bars for the observed give 95% confidence intervals, assuming a Poisson distribution of the number of dead females and males in each time interval, and the error bars for the expected are 2.5% and 97.5% quantiles for the MCMC posterior distribution of the expected number of dead females and males per vial in each time interval

## Appendix 1

### Bayesian MCMC Analysis of ‘Time‐to‐Event’ Data

Data on survival and cold‐shock coma recovery consisted of counts of dead or recovered flies in vials in a number of time intervals of varying length. Our aim was to analyze differences between the selection regimes (R, U, and C) and effects of cold shock, starvation, and sex, at the same time accounting for random variation among replicate lines and vials. The data files and R and Stan code files used for the analysis are deposited at the Dryad Repository (doi:10.5061/dryad.4790c).

Here, we give an overview of the analysis of starvation survival (Figure 2a–c; Table 1; Table A1; Figure A1). We modeled the survival time under starvation in the same way as is commonly done in Bayesian approaches to mixed‐effect modeling of quantitative response variables (e.g., in the stan_lmer function in the R package rstanarm; Gabry & Goodrich, 2016), in that the survival time was decomposed into fixed effects and normally distributed random effects. From the posterior distribution of survival times, we then determined the probability of death for females and males in each time interval and assumed that the observed number of dead females and males were multinomially distributed based on these probabilities. An examination of how well observed and predicted matched (often referred to as a posterior predictive check) appears in Figure A1.

**Table A1:** Bayesian MCMC model output for the number of hours of starvation survival, estimated in generation 14

Effect	Estimate	95% C.I.
σ_F_	19.07	(18.65, 19.53)
σ_M_	10.84	(10.55, 11.12)
σ_vlF_	4.28	(3.44, 5.24)
σ_vlM_	2.51	(1.97, 3.08)
σ_cuFM_	1.82	(0.21, 3.72)
σ_cuF_	0.92	(0.04, 2.61)
σ_cuM_	1.35	(0.07, 3.15)
σ_rpFM_	1.16	(0.10, 2.43)
σ_rpF_	2.23	(0.36, 4.18)
σ_rpM_	0.72	(0.03, 1.92)
Intercept	104.64	(101.79, 107.44)
Sex M	−25.80	(−28.78, −22.92)
Sel U	−3.89	(−7.81, −0.23)
Sel C	−17.92	(−21.66, −14.23)
Cue Cs	−7.56	(−10.26, −4.88)
Age D_2‐3_	−10.95	(−17.63, −4.02)
Age D_3_	5.62	(4.00, 7.17)
Sex M × Sel U	1.30	(−2.34, 5.07)
Sex M × Sel C	1.17	(−2.44, 4.99)
Sex M × Cue Cs	0.60	(−1.64, 2.67)
Sex M × Age _D2‐3_	6.36	(−0.83, 13.55)
Sex M × Age D_3_	−9.32	(−11.32, −7.35)
Sel U × Cue Cs	1.43	(−1.96, 5.02)
Sel C × Cue Cs	3.58	(0.18, 6.85)

The intercept represents females (F) from selection regime R without cold shock, and age 2 days. The table gives estimates and confidence intervals for all parameters in the model

The model included a number of variance components (see Table A1 for estimates of all parameters in the model), including the standard deviation of the starvation survival for females and males within a vial (σ_F_ and σ_M_), between vials within a replicate line and cold‐shock cue treatment (σ_vlF_ and σ_vlM_), between cues within replicate line (σ_cuFM_, σ_cuF_, and σ_cuM_, where the fist component allows for a possible female–male correlation), and between replicates lines within selection regimes (σ_rpFM_, σ_rpF_, and σ_rpM_). The fixed effects in Table A1 include sex (Sex M), selection regime (Sel U, Sel C), cold shock cue (Cue Cs), age (Age D_2‐3_, Age D_3_), and their interactions.
